# Learning From the Use of Generative AI in Fourth-Year Psychiatry Teaching at Aston Medical School

**DOI:** 10.1192/bjo.2026.11352

**Published:** 2026-06-30

**Authors:** Shahnoor Adil, Ghaniah Hassan-Smith, Shagaf Bakour, Anis Ahmed, Priyanka Sharma

**Affiliations:** Aston Medical School, Birmingham, United Kingdom

## Abstract

**Aims:**

Undergraduate psychiatry education is frequently limited by variable clinical exposure, ethical constraints in involving acutely unwell patients, and restricted opportunitiesfor students to practise high-stakes consultations. Generative artificial intelligence (AI) simulation platforms offer a potential solution by enabling students to rehearse clinical encounters in a structured and safe environment. This poster aims to present lessons learned from the implementation of generative AI-supported simulations for fourth-year medical students at Aston Medical School. Specifically, it explores how students and tutors experienced the integration of AI-facilitated consultations into the curriculum and identifies factors that influenced engagement, learning value, and trust in assessment.

**Methods:**

A mixed-methods educational evaluation was conducted following the introduction of AI-based consultation simulations within the fourth-year psychiatry programme. Feedback was gathered through anonymised student questionnaires, facilitated debrief sessions after simulation use, and structured discussions with clinical tutors involved in teaching and assessment. Data focused on students’ perceived learning value, alignment with clinical placements, clarity of feedback and marking criteria, and overall usability of the platform. Tutor reflections on feasibility, integration with existing teaching, and impact on small-group learning were also collected. Qualitative themes were identified through thematic review of student and tutor comments.

**Results:**

Students generally recognised the potential value of generative AI simulation as a safe environment to practise structured consultations, particularly for OSCE preparation and rehearsal of high-risk scenarios such as suicide risk assessment and capacity evaluations. Many reported that the tool was most effective when used after formal teaching or placement exposure, supporting consolidation and revision. However, engagement was reduced when simulations were used as a first exposure to unfamiliar clinical material or were misaligned with placement timing. Some students expressed uncertainty about AI-generated markingcriteria and pass thresholds, noting that checklist-based feedback could sometimes feel disconnected from authentic clinical reasoning. Mandatory debrief sessions were valued when they facilitated reflection and discussion but were perceived as less helpful when they repeated automated feedback. Tutors highlighted the importance of clear learning objectives, integration with small-group teaching, and transparency around assessment processes.

**Conclusion:**

Generative AI simulation can enhance undergraduate psychiatry education by providing scalable opportunities for structured practice and feedback. Its effectiveness depends on thoughtful curriculum integration, alignment with clinical teaching, and clarity around assessment and feedback. Student and tutor perspectives suggest that AI is most beneficial when used to augment, rather than replace, traditional teaching and reflective debriefing. These lessons offer practical insights for educators seeking to incorporate generative AI into medical education in a pedagogically meaningful and learner-centred way.

